# Corticosteroid responsiveness in patients with acute exacerbation of interstitial lung disease admitted to the emergency department

**DOI:** 10.1038/s41598-021-85539-1

**Published:** 2021-03-11

**Authors:** Hye Jin Jang, Seung Hyun Yong, Ah Young Leem, Su Hwan Lee, Song Yee Kim, Sang Hoon Lee, Eun Young Kim, Kyung Soo Chung, Ji Ye Jung, Young Ae Kang, Young Sam Kim, Joon Chang, Moo Suk Park

**Affiliations:** Division of Pulmonology, Department of Internal Medicine, Severance Hospital, Yonsei University College of Medicine, 50-1 Yonsei-ro, Seodaemun-gu, Seoul, 03722 Republic of Korea

**Keywords:** Diseases, Medical research

## Abstract

Interstitial lung disease (ILD), particularly idiopathic pulmonary fibrosis (IPF), has a poor prognosis. Corticosteroids are widely used in the treatment of acute exacerbation of ILD (AE-ILD). This study aimed to clarify the causes of AE-ILD, determine the efficacy of corticosteroids for treating AE-ILD, and detect differences in the mortality rate among subgroups of ILD. This was an observational retrospective single-center study. Patients with ILD who presented to the emergency department with acute respiratory symptoms from January 1, 2016, to December 31, 2018, were included. Patients with AE-ILD were classified into two groups depending on the prednisolone dose: low dose (0 to 1.0 mg/kg) or high dose (> 1.0 mg/kg). Mortality rates between patients with and without IPF were compared. This study included 182 patients with AE-ILD, including IPF (n = 117) and non-IPF (n = 65). Multivariate Cox regression analysis showed that corticosteroid dose (HR: 0.221, CI: 0.102–0.408, *P* < 0.001), initial P/F ratio (HR:0.995, CI:0.992–0.999, *P* = 0.006), and mechanical ventilation within 3 days of hospitalization (HR:4.205, CI:2.059–8.589, *P* < 0.001) were independent risk factors for mortality in patients with AE-ILD. This study showed that outcomes improve with higher doses of corticosteroids (> 1 mg/kg prednisolone) in patients with AE-non-IPF-ILD. However, this was not the case in patients with AE-IPF.

## Introduction

Interstitial lung disease (ILD) is a diffuse parenchymal lung disease characterized by inflammation or fibrosis of the interstitium, alveoli, or terminal bronchioles, with etiologies ranging from rheumatologic or environmental to idiopathic^1^^.^ It has been estimated that approximately 100,000 hospital admissions per year and 15% of all pulmonologist appointments are due to ILD^2^^.^ The common feature across different ILD types, irrespective of the clinical presentation, disease progression, or prognosis, is fibrotic destruction of the lung parenchyma^3^^.^ An acute exacerbation of ILD (AE-ILD) can occur at any time, and is associated with significant morbidity and mortality.

Idiopathic pulmonary fibrosis (IPF) is the most severe form of ILD. Acute exacerbation of IPF (AE-IPF) portends an even worse prognosis, with earlier studies reporting a mortality of 50–80% and a median survival time of 3–4 months^4,5^^.^ However, more recent studies have suggested slightly higher survival rates: 1-month survival rate of 66% (range: 47–85%); 3-month survival rate of 41% (range: 0–54%); and survival to hospital discharge of 44% (4–77%)^6^^.^ A study conducted in Japan has even reported survival rates of over 70% in patients treated with cyclosporin A or high-dose corticosteroids^7^^.^ Approximately 20% of patients with IPF will experience AE-IPF over the course of their disease, and about one-fifth of these patients will have more than one AE-IPF episode^4–6^; thus, AE is now recognized as a relatively common event^7^^.^ Although AE has been mainly described in patients with IPF, the presentation and consequences of AE appear to be similar in other ILDs. While there is no cohesive definition of AE for ILDs other than IPF, criteria similar to those of AE-IPF are often used^1^^.^ Additionally, AE-ILD has been associated with decreased survival in patients with any connective tissue disease-related ILD^8^^.^ Although AE-IPF is being increasingly recognized and perceived as a severe event with high associated mortality, there is only a limited amount of clinical data on AE-ILD in non-IPF ILD^9^^.^ Some studies have attempted to identify risk factors common to all forms of AE-ILD; however, their results have often been inconsistent^1^^.^ In IPF, low forced vital capacity (FVC), low total lung capacity, decreased 6-min walk distance, pulmonary hypertension, baseline hypoxemia, low diffusing capacity of the lung for carbon monoxide (DL_CO_), increased dyspnea, and no history of smoking are all associated with an increased risk of exacerbation^10,11^^.^ Nevertheless, previous sufficient case series have failed to confirm these associations.

High-dose corticosteroids, sometimes administered as pulse therapy (with or without immunosuppressive agents) in combination with broad-spectrum antimicrobial agents, have been used most commonly in patients with AE-ILD. This is the recommended treatment according to recent international treatment guidelines for IPF^12^^.^ However, the efficacy of this therapy is uncertain, and survival is still very low^4^^.^

The aim of this study was to clarify the causes of AE-ILD and the risk factors associated with mortality, particularly corticosteroid responsiveness, in patients with AE-ILD admitted to the emergency department (ED).

## Results

### Patients recruitment

**Figure 1:**
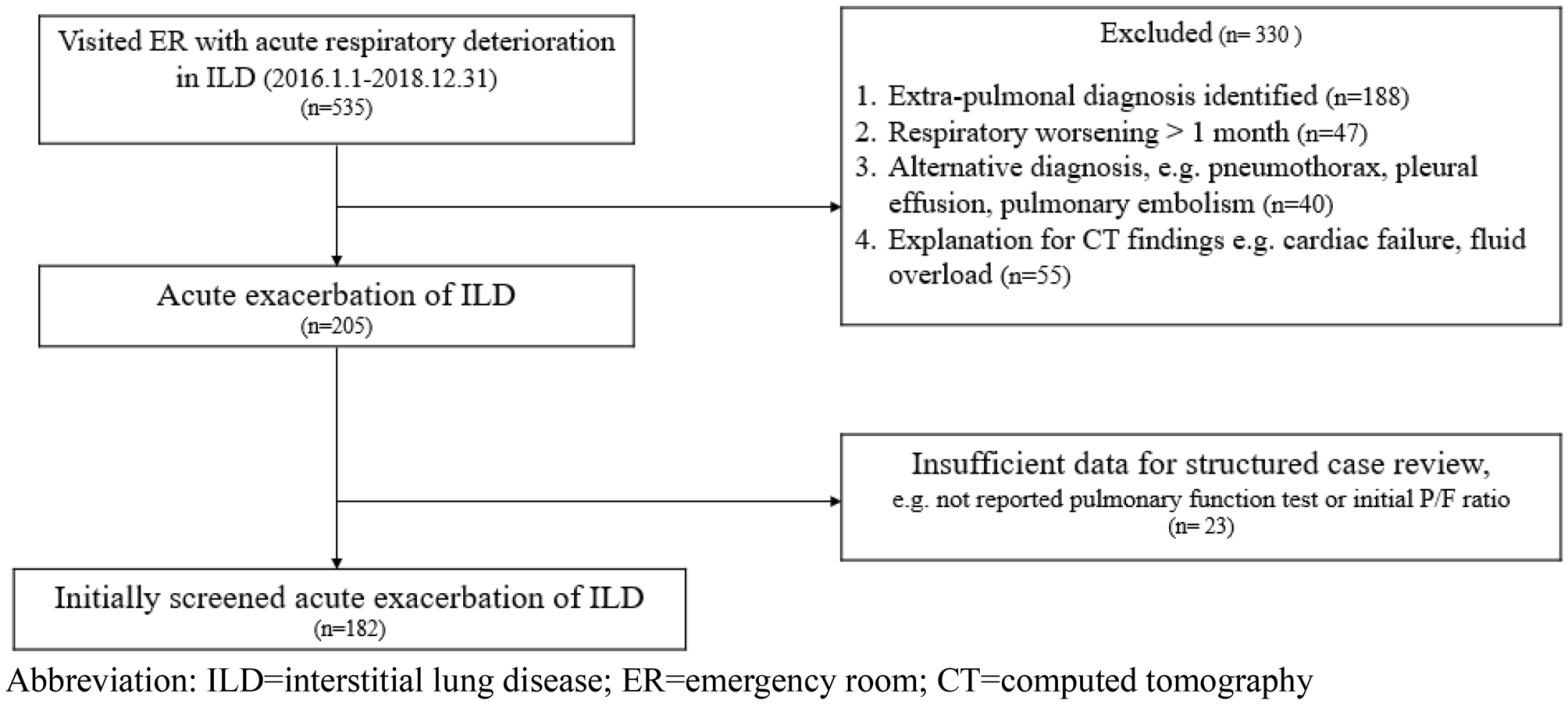
Patient recruitment flowchart. *ILD* interstitial lung disease, *ER* emergency room, *CT* computed tomography.

We reviewed medical records and all chest computed tomography (CT) scans of patients who were diagnosed with ILD and presented to the ED with symptoms of dyspnea at the Severance Hospital between January 2016 and December 2018. A total of 535 patients were initially screened and 330 were excluded for the following reasons: (1) fluid overload, (2) congestive heart failure, (3) pulmonary embolism, (4) dyspnea unrelated to respiratory disease, and (5) worsening symptoms for more than 1 month. Finally, 205 patients with AE-ILD were enrolled. A further 23 cases were excluded because of insufficient data, including lack of pulmonary function test results within 6 months before the ED visit and unavailable baseline arterial blood gas values, resulting in 182 selected patients. In June 2019, all subjects were reevaluated by multidisciplinary discussion using patient history, physical examination, laboratory results, chest high-resolution CT (HRCT), and histopathological findings. The final diagnoses were categorized as IPF and non-IPF ILD; the latter diagnosis included connective tissue disease-associated ILD, idiopathic nonspecific interstitial pneumonia, cryptogenic organizing pneumonia, sarcoidosis, hypersensitivity pneumonitis, and other conditions (toxic humidifier sterilizers-induced ILD and amiodarone-induced ILD). Among the included patients, 117 had IPF and 65 had non-IPF ILD (Fig. 1).

### Patient characteristics and microbiology

A total of 182 patients were included in the study, and the baseline characteristics of the study sample are shown in Table 1. The mean patient age was 68.7 ± 11.6 years. Among the total patients, 117 (64.3%) had IPF, 67.0% were male, and 45.4% had a history of smoking. Patients in the IPF group were more commonly exposed to smoking (57.9%) than those in the non-IPF ILD group (21.7%). FVC and forced expiratory volume in the first second (FEV_1_) were significantly higher in the non-IPF ILD group compared to the IPF group.

**Table 1:** Baseline characteristics of patients with acute exacerbation of IPF and non-IPF ILD.

Variable	Total	IPF	Non-IPF	*P*-value
Total patients	182	117	65	
Age, years	68.7 ± 11.6	69.4 ± 9.9	67.4 ± 14.1	0.27
Sex, men	122 (67.0)	94 (80.3)	28 (43.1)	< 0.001
**Smoking exposure, n (%)**				
Never	95 (54.6)	48 (42.1)	47 (78.3)	< 0.001
Former	77 (44.3)	64 (56.1)	13 (21.7)	
Current	2 (1.1)	2 (1.8)	0 (0.0)	
Pack-years	35.0 (20.0–48.5)	35.0 (20.0–50.0)	25.0 (20.0–40.0)	0.09
FVC % predicted	60.0 (46.0–74.0)	57.0 (41.0–69.0)	67.0 (55.0–80.0)	0.003
FEV_1_% predicted	71.0 (56.0–85.0)	67.0 (50.5–80.0)	75.5 (61.0–92.0)	0.02
DL_CO_, % predicted	47.0 (35.0–59.0)	44.0 (31.0–60.0)	49.0 (37.3–58.0)	0.11
Initial P/F ratio	223.4 (158.0–310.0)	220.0 (148.0–291.7)	233.9 (179.6–339.3)	0.29
GAP score	4 (4.0, 6.0)	4 (3.0, 4.0)	4 (3.75, 5.0)	0.16
Prednisolone (mg/kg)	1.0 ± 0.9	1.0 ± 0.8	1.1 ± 0.9	0.20
Previous AE history	60 (33.0)	43 (36.8)	17 (26.2)	0.20
Anti-fibrotics	54 (29.7)	54 (46.2)	0 (0.0)	< 0.001
Supplemental O_2_	37 (20.3)	28 (23.9)	9 (13.8)	0.19
Prednisolone before AE	91 (50.0)	49 (41.9)	42 (64.6)	0.97
**Medical history, n (%)**				
Hypertension	33 (18.1)	25 (21.4)	8 (12.3)	0.13
Diabetes mellitus	32 (17.6)	24 (20.5)	8 (12.3)	0.16
CAOD	19 (10.4)	13 (11.1)	6 (9.2)	0.69
NTM	7 (3.8)	4 (3.4)	3 (4.6)	0.69
Old TB	11 (6.0)	8 (6.8)	3 (4.6)	0.55
COPD	12 (6.6)	6 (5.1)	6 (9.2)	0.29
Malignancy	36 (19.8)	21 (17.9)	15 (23.1)	0.41
Use of vasopressors within 3 days	26 (14.3)	19 (16.2)	7 (10.8)	0.31
Need for mechanical ventilator	35 (19.2)	24 (20.5)	11 (16.9)	0.56
CRP (mg/L)	70.5 (21.0–140.0)	65.0 (21.0–136.0)	78.0 (27.0–140.0)	0.33
90 day-mortality	40 (22.0)	30 (25.6)	10 (15.4)	0.21

Data are presented as mean and standard deviation, median and interquartile range, or frequency (%).

*IPF* idiopathic pulmonary fibrosis, *ILD* interstitial lung disease, *FVC* forced vital capacity, *FEV*_*1*_ forced expiratory volume in 1 s, *DLco* diffusing capacity of carbon monoxide, *P/F ratio* partial pressure of oxygen in arterial blood (PaO_2_)/fraction of inspired oxygen (FiO_2_) ratio, *GAP score system* gender (G), age (A), physiology (P), *AE* acute exacerbation, *CAOD* coronary artery occlusive disease, *NTM* non-tuberculous mycobacterium, *COPD* chronic obstructive lung disease, *Old TB* previous tuberculosis, *CRP* C-reactive protein.

The mean dose of prednisolone was 1.0 mg/kg in the IPF group and 1.1 mg/kg in the non-IPF ILD group. The total mortality was 40 cases (22.0%), with 30 cases (25.6%) in the IPF group and 10 cases (15.4%) in the non-IPF group.

Table 2 shows the comparison between the triggered AE-ILD and non-triggered AE-ILD groups. The triggered AE-ILD group had lower P/F ratios and higher prednisolone doses and lactate levels. The mortality was 11 cases (22%) in the triggered group and 12 cases (15.8%) in the non-triggered group; however, the difference was not statistically significant (*P* = 0.377).

**Table 2:** Comparison of baseline characteristics between triggered and non-triggered AE-ILD groups.

Variable	Triggered group	Non-triggered group	*P*-value
Total patients, n	50	81	
Age, years	71.5 (60.8, 77.0)	72 (64.0, 78.0)	0.271
Sex, male	34 (68)	45 (55.6)	0.318
IPF	31 (62)	42 (51.9)	0.454
Initial P/F ratio	217.2 (160.3, 285.8)	253.6 (188.7, 400.0)	0.017
FVC (%), predicted	60 (47.0, 73.0)	62.0 (51.5, 78.5)	0.623
DLco (%), predicted	50.0 (33.3, 66.8)	49.0 (36.8, 58.3)	0.749
Prednisolone (mg/kg)	1.1 (0.5, 1.5)	0.6 (0.2, 1.2)	0.011
CRP (mg/L)	68.5 (28.5, 145.5)	78.5 (28.4, 159.8)	0.437
Lactate (mmol/L)	1.6 (1.3, 2.7)	1.4 (1.0, 1.9)	0.024
Use of vasopressors within 3 days	7 (14)	15 (19.7)	0.407
Need for mechanical ventilator	11 (22)	24 (29.6)	0.402
90-day mortality	11 (22)	12 (15.8)	0.377

Data are presented as mean and standard deviation, median and interquartile range, or frequency (%).

*IPF* idiopathic pulmonary fibrosis, *AE-ILD* acute exacerbation of interstitial lung disease, *FVC* forced vital capacity, *DLco* diffusing capacity of carbon monoxide, *P/F ratio* partial pressure of oxygen in arterial blood (PaO_2_)/fraction of inspired oxygen (FiO_2_) ratio, *CRP* C-reactive protein.

Table 3 shows the microbiologic analysis of the triggered AE-ILD group. Bacterial infection was dominant in both IPF (23.4%) and non-IPF (15.2%) ILD groups. Viral infection was the second most common trigger in the IPF group, while fungal infection (e.g. *Pneumocystis jiroveci* pneumonia) was the second most common trigger in the non-IPF ILD group (13.5%).

**Table 3:** Microbiologic results in the triggered AE-ILD group.

Microbiology, *n*	Total (n = 131)	IPF (n = 74)	Non-IPF ILD (n = 57)
Negative results, %	81 (61.8)	46 (62.2)	35 (61.4)
Bacterial infection, %	28 (21.4)	18 (24.3)	10 (17.5)
*Pseudomonas aeruginosa*	7 (5.3)	5 (6.8)	2 (3.5)
*Mycoplasma*	6 (4.6)	5 (6.8)	1 (1.8)
*Legionella*	6 (4.6)	4 (5.4)	2 (3.5)
*Klebsiella pneumoniae*	5 (3.8)	2 (2.7)	3 (5.4)
*Stenotrophomonas maltophilia*	1 (0.7)	1 (1.4)	0 (0)
*Streptococcus pneumoniae*	1 (0.7)	1 (1.4)	0 (0)
*Acinetobacter baumannii*	1 (0.7)	0 (0)	1 (1.8)
*Serratia marcescens*	1 (0.7)	0 (0)	1 (1.8)
Viral infection^a^, %	10 (7.0)	7 (9.5)	3 (5.4)
Fungal infection, %	12 (9.2)	3 (4.2)	9 (15.8)
*Pneumocystis jiroveci*	11 (8.4)	2 (2.7)	9 (15.8)
*Aspergillus*	1 (0.7)	1 (1.4)	0 (0)

*AE-ILD* acute exacerbation of interstitial lung disease.

^a^Metapneumovirus, Influenza virus, Coronavirus, Rhinovirus, and RSV.

### Survival outcomes

Table 4 shows the comparison of characteristics between survivors and non-survivors. Forty (22%) patients out of 182 died within 90 days, and most were male (70%). The non-survivor group showed a trend of lower FEV_1_, DLco, and P/F ratio. Prednisolone was prescribed at a higher dose in the survivor group (1.1 ± 0.9) than in the non-survivor group (0.9 ± 0.6), but this was not statistically significant. The rate of mechanical ventilation was more than three-fold higher in the non-survivor group (42.5%) than in the survivor group (12.7%), and this was statistically significant. The use of vasopressors in the first 3 hospitalization days was higher in the non-survivor group (22.5% compared to 12.0% in the survivor group). Comparisons between survivors and non-survivors within the IPF group (e-Table 1) and within the non-IPF group (e-Table 2) yielded equivalent results.

**Table 4:** Comparison of characteristics between survivors and non-survivors in patients with acute exacerbation of ILD.

Variable	Total	Survivors	Non-survivors	*P*-value
Total patients	182	142 (78.0)	40 (22.0)	
Age, years	68.7 ± 11.6	69.0 ± 11.7	67.6 ± 11.5	0.52
Sex, men	122 (67.0)	94 (66.2)	28 (70.0)	0.65
**Smoking exposure, no. (%)**				
Never	95 (54.6)	72 (53.3)	23 (59.0)	0.65
Former	77 (44.3)	61 (45.2)	16 (41.0)	
Current	2 ( 1.1)	2 ( 1.5)	0 ( 0.0)	
Pack-years	35.0 (20.0–48.5)	35.0 (20.0–50.0)	35.0 (18.0–40.0)	0.38
FVC % predicted	60.0 (46.0–74.0)	60.0 (47.0–75.0)	60.0 (45.0–70.0)	0.52
FEV_1_% predicted	71.0 (56.0–85.0)	72.0 (56.0–85.0)	68.0 (52.0–80.0)	0.43
DL_CO_, % predicted	47.0 (35.0–59.0)	49.0 (37.0–61.5)	36.5 (29.5–54.0)	0.14
Initial P/F ratio	223.4 (158.0–310.0)	236.4 (158.9–316.7)	210.5 (149.6–279.0)	0.32
GAP score	4 (4.0, 6.0)	4 (3.75, 5.0)	4 (3.0, 4.0)	0.16
Prednisolone (mg/kg)	1.0 ± 0.9	1.1 ± 0.9	0.9 ± 0.6	0.45
Previous AE history	60 (33.0)	48 (33.8)	12 (30.0)	0.59
Anti-fibrotics	54 (29.7)	46 (32.4)	8 (20.0)	0.87
Supplemental O_2_	37 (20.3)	29 (20.4)	8 (20.0)	0.29
Prednisolone before AE	91 (50.0)	73 (51.4)	18 (45.0)	0.29
**Medical history, n (%)**				
Hypertension	33 (18.1)	23 (16.2)	10 (25.0)	0.20
Diabetes mellitus	32 (17.6)	27 (19.0)	5 (12.5)	0.34
CAOD	19 (10.4)	17 (12.0)	2 (5.0)	0.20
IPF	117 (64.3)	87 (61.3)	30 (75.0)	0.11
NTM	7 (3.8)	6 (4.2)	1 (2.5)	0.62
Old TB	11 (6.0)	10 (7.0)	1 (2.5)	0.29
COPD	12 (6.6)	11 (7.7)	1 (2.5)	0.24
Malignancy	36 (19.8)	27 (19.0)	9 (22.5)	0.62
CRP (mg/L)	70.5 (21.0–140.0)	62.4 (21.0–139.0)	81.5 (35.0–144.5)	0.22
Need for mechanical ventilator	35 (19.2)	18 (12.7)	17 (42.5)	< 0.001
Use of vasopressors within 3 days	26 (14.3)	17 (12.0)	9 (22.5)	0.09

Data are presented as mean and standard deviation, median and interquartile range, or frequency (%).

*ILD* interstitial lung disease, *FVC* forced vital capacity, *FEV*_*1*_ forced expiratory volume in 1 s, *DLco* diffusing capacity of carbon monoxide, *P/F ratio* partial pressure of oxygen in arterial blood (PaO_2_)/fraction of inspired oxygen (FiO_2_) ratio, *GAP score system* gender (G), age (A), physiology (P), *AE* acute exacerbation, *CAOD* coronary artery occlusive disease, *IPF* idiopathic pulmonary fibrosis, *NTM* non-tuberculous mycobacterium, *COPD* chronic obstructive lung disease, *Old TB* previous tuberculosis, *CRP* C-reactive protein.

Table 5 shows the risk factors related to mortality, which were initial P/F ratio (HR: 0.995, CI: 0.992–0.999, *P* = 0.006), high-dose of prednisolone (HR: 0.221, CI: 0.102–0.480, *P* < 0.001), and need for mechanical ventilation in the first 3 hospitalization days (HR: 4.205, CI: 2.059–8.589, *P* < 0.001).

**Table 5:** Cox regression analysis of risk factors related to 90-day mortality in AE-ILD.

Variable	Univariate			Multivariate		
	HR	95% CI	p-value	HR	95% CI	p-value
Age, years	0.993	0.968–1.019	0.603	0.989	0.957–1.009	0.200
Sex, male	1.228	0.624–2.415	0.552	0.777	0.377–1.599	0.493
Initial P/F ratio	0.998	0.998–1.001	0.241	0.995	0.992–0.999	0.006
FVC (%), predicted	0.994	0.975–1.014	0.540			
DLco (%), predicted	0.976	0.952–1.001	0.059			
Prednisolone > 1 mg/kg	0.380	0.193–0.747	0.005	0.221	0.102–0.480	< 0.001
Use of vasopressors within 3 days	1.852	0.881–3.890	0.104	1.451	0.630–3.340	0.382
Need for mechanical ventilator	3.877	2.068–7.267	< 0.001	4.205	2.059–8.589	< 0.001

*AE-ILD* acute exacerbation of interstitial lung disease, *FVC* forced vital capacity, *DLco* diffusing capacity of carbon monoxide, *P/F ratio* partial pressure of oxygen in arterial blood (PaO_2_)/fraction of inspired oxygen (FiO_2_) ratio, *HR* hazard ratio.

**Figure 2:**
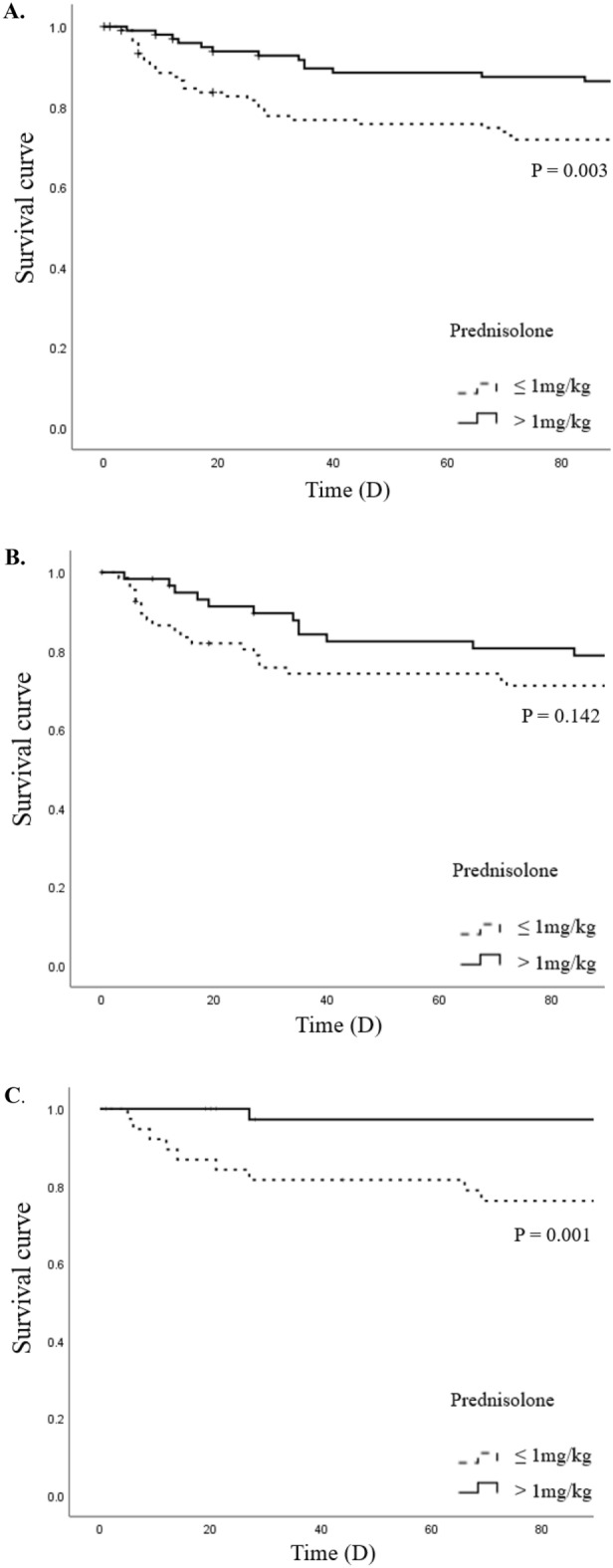
Kaplan–Meier survival curves according to corticosteroid use in (**A**) total patients; (**B)** patients with IPF; (**C)** patients with non-IPF ILD.

Kaplan–Meier estimates of 90-day mortality according to corticosteroid dose are shown in Fig. 2.

The survival rate was significantly higher when the prednisolone dose was higher than 1 mg/kg in the total sample of AE-ILD patients (Fig. 2A, log-rank test, *P* = 0.003). The same results were observed in the non-IPF ILD group (Fig. 2C, log-rank test, *P* = 0.001). In the IPF group, however, the 90-day mortality analysis was not statistically significant (Fig. 2B, log-rank test, *P* = 0.142). e-Fig. 1 shows that survival rates of the triggered and non-triggered AE-ILD groups according to corticosteroid dose were not significantly different. The main cause of death was respiratory failure caused by AE-ILD (n = 24), followed by pneumonia (n = 10) and combined lung cancer progression (n = 6).

## Discussion

This retrospective study showed that high doses of corticosteroids are beneficial in most cases of AE-ILD, especially in cases of AE-non-IPF ILD, but not in cases of AE-IPF. Survival improved when doses of prednisolone higher than 1 mg/kg were administered.

The immediate outcome of AE is very poor; the median survival is 2.2 months from onset, and half of the patients die during hospitalization^4,6^^.^ Furthermore, about 50% of patients need admission to an intensive care unit^4^^.^ However, the optimal treatment for AE-IPF remains undetermined. A recent retrospective study showed that pirfenidone combined with corticosteroids and recombinant human thrombomodulin may improve survival in patients with AE-IPF (55% in patients with pirfenidone vs 34% in control group, p = 0.042)^13^. Most experts^14^ administer systemic corticosteroids with or without immunosuppressants, but the various drug regimens used in these two treatment approaches have not been evaluated in randomized controlled trials. Hence, the recommendation of corticosteroid treatment is based on low-quality evidence^12^^.^ However, most patients are treated with high-dose immunosuppression therapy, typically with pulses of 500–1000 mg of methylprednisolone daily for 3 days^3,6^^.^ The 2018 Japanese IPF treatment guidelines suggest that patients with AE-IPF should be treated with corticosteroids, including pulse therapy^15^^.^ This is contradicted by the results of Mengshu et al.^16^ who reported that high doses of corticosteroids are not beneficial for AE-ILD patients, and that the clinical outcomes of patients with AE-IPF mainly depend on the underlying clinical condition and the extent of lung injury from the AE. Thus, there remains a lack of consensus regarding proper treatment of AE-ILD.

Feghali-Bostwick et al.^17^ showed that patients with IPF frequently have autoantibodies and self-reactive CD4 T cells, thus fulfilling the diagnostic criteria for an autoimmune disease^18^^.^ Moreover, there is a component of inflammation in AE-IPF^19^^.^ Therefore, some patients respond to corticosteroids^20^^.^ However, a recent study from 2020 showed no evidence that corticosteroids improve the outcome of patients with AE-IPF admitted to the hospital; furthermore, the results indicated that corticosteroid use following an exacerbation may contribute to reduced overall survival^21^^.^ Our study showed the same results in AE-IPF cases.

The 90-day mortality rate in this study was relatively low (25.6% in the IPF group and 15.4% in the non-IPF ILD group) compared to other studies (mortality rate: 50%–80%)^10,22^. Initial P/F ratio was a statistically significant predictor of mortality in this study, with a lower P/F ratio predicting a higher mortality rate. The patients in our study were mainly diagnosed with mild ARDS (median P/F ratio: 223.4), and pirfenidone was administered in 41.6% (n = 54) of IPF patients before AE. A higher survival rate has been reported for patients with AE-IPF who were administered pirfenidone (55%) compared to those not administered pirfenidone (34%; p = 0.042)^13^. Recent developments in ARDS and sepsis management, as well as the use of high corticosteroid doses, may contribute to reduced mortality rates in this patient group.

Non-IPF ILD is also associated with autoimmunity, and thus has potential to respond well to corticosteroid treatment. According to the abovementioned mechanism of ILD, it could be speculated that corticosteroids would be effective in cases of AE-non-IPF ILD, but not in cases of AE-IPF.

AE-ILD can be precipitated by a variety of factors^1^^.^ Viral infections, air pollution, aspiration, transfusions, drugs, or surgery, all leading to acceleration of the fibro-proliferative response, have been postulated as triggers of AE-ILD. However, the specific factors that cause some patients with ILD and not others to develop AE are unknown^3,23^^.^ Although an infectious trigger could explain why some patients develop AE and others do not, no firm associations between bacterial or viral infections (including Epstein-Barr virus, cytomegalovirus, and human herpesvirus 8) and AE have been identified^6^^.^ In our study, patients were classified as triggered and non-triggered AE-ILD based on their bacterial, fungal, or viral specimens within 48 h of ED visit. While samples could not be collected from 51 patients, 131 out of 182 patients met the above conditions. Our study identified 50 cases of AE-ILD triggered by bacterial, fungal, or viral infections. These patients had a worse survival outcome (90-day mortality of 22% compared to 15.8% in the non-triggered AE cases), although the difference was not statistically significant. However, triggered AE-ILD cases showed a more severe clinical course, including lower P/F ratios and higher lactate levels. Depending on the severity of the disease, fluid resuscitation, antibiotic use, initial ventilator care, sepsis management, and corticosteroid therapy could be needed.

Huie et al. suggested that patients with IPF have a lower survival rate after an acute respiratory decline as compared to patients with non-IPF fibrotic disease^24^^.^ Pathophysiologically, AE-ILD resembles an acute lung injury, which presents histopathologically as diffuse alveolar damage in most cases^25^^.^ A number of factors (including low baseline FVC and DL_CO_, extensive CT abnormalities and the involvement pattern, low oxygenation, and high bronchoalveolar lavage fluid neutrophil and lymphocyte percentages at the time of AE) have been reported as associated with survival of patients with AE-IPF^3,26,27^^.^ However, this study could not demonstrate the significance of the radiologic and cellular pattern of bronchoalveolar lavage fluid because of a small sample size.

Patients who required mechanical ventilation from the beginning had a significantly higher mortality rate, possibly due to individual differences in the response to oxygen and steroid therapy in the setting of AE. Further studies are needed to assess individual differences in the response to oxygen and steroid therapy.

The present study had several limitations. First, this study analyzed data of a single tertiary center. Second, although we tried to obtain complete data by reviewing medical records, not all patients underwent a complete examination (e.g., missing microbiologic studies) at the initial visit to the ED. Third, we could not analyze long-term complications of corticosteroid therapy; therefore, we could not identify whether their use led to adverse outcomes such as opportunistic infections.

Despite the limitations, this study provides useful evidence for proper initial management and for decision-making regarding corticosteroid use in patients with AE-non-IPF ILD. Determining the corticosteroid dose and the administration modality (pulse therapy or routine high-dose steroids) is always difficult because of the potential side effects of corticosteroids. A larger-sample prospective study of patients with AE-ILD is, therefore, needed to address these limitations.

## Methods

### Study design and sample

This study was conducted retrospectively in a tertiary care hospital in South Korea.

The following clinical and laboratory data were collected from medical records: age, sex, smoking history, pulmonary function test results within 6 months before presenting to the ED, underlying diseases, ratio of initial partial pressure of oxygen in arterial blood (PaO_2_) to fraction of inspired oxygen (FiO_2_; P/F ratio), gender-age-physiology (GAP) index score, prednisolone dose, serum concentrations of C-reactive protein (CRP) and lactate, and date of death. An ever smoker was defined as a person who had smoked at least 100 cigarettes or cigars during the course of their life.

The research protocol was approved by the Institutional Review Board of Severance Hospital, South Korea (IRB No. 4–2020-0398). The study design was approved by the appropriate ethics review boards. The requirement to obtain informed patient consent was waived by the IRB.

### Definitions

In this study, AE-IPF was defined as a clinically significant respiratory deterioration developing typically within less than 1 month, accompanied by new radiologic abnormalities on HRCT (e.g., diffuse bilateral ground-glass opacification), and with an absence of other obvious clinical causes such as fluid overload, congestive heart failure, or pulmonary embolism. This is in accordance with the official American Thoracic Society/European Respiratory Society/Japanese Respiratory Society/Latin American Thoracic Society IPF guidelines^10^^.^

This new definition promotes discrimination between a triggered form of AE-IPF (e.g., infectious, postprocedural/postoperative, or toxic) and an idiopathic form of AE-IPF, in which no trigger is identified^5,10^. Regarding other AE-ILDs, it is more challenging to establish the specific diagnoses. The most important element for diagnosing AE-ILDs is the HRCT, which must be performed when patients are clinically stable. The typical HRCT findings in AE-ILD are newly developed bilateral alveolar infiltrates and ground-glass opacification with or without consolidation^6,12^^.^ Since there is no definition of AE in non-IPF ILD cases, the definition of AE-IPF was applied.

The GAP score was calculated for all patients (IPF and non-IPF) based on sex (0–1 points), age (0–2 points), FVC (0–2 points), and DL_CO_ (0–3 points), and classified into stages I (0–3 points), II (4–5 points), or III (6–8 points), as previously described^28^^.^ Outcome measures were determined based on the medical record review. The primary outcome measure was 90-day mortality.

### Treatment protocol and steroid dosage

For patients diagnosed with AE-ILD in the ED, blood tests (including serum inflammatory markers and routine chemistry), microbiologic cultures, and chest CT scans were initially performed. Bronchoscopy with bronchoalveolar lavage, which is an invasive diagnostic procedure, was not routinely performed initially due to poor patient tolerance.

In our institution, the main treatment for AE-ILD is the administration of corticosteroids together with effective antibiotics. Additionally, we aim for symptom palliation with supplemental oxygen therapy, mechanical ventilation when needed, acute respiratory distress syndrome (ARDS) therapy, and shock management.

Corticosteroids are usually administered at doses ranging from 0.5 to 2 mg/kg, depending on the clinical situation. Steroid pulse therapy was not usually applied except for cases of vasculitis or dermatomyositis-associated rapid progressive ILD.

### Statistical analysis

Baseline characteristics, including demographics, baseline pulmonary function, respiratory parameters, hematologic data, serum CRP and lactate, and survival, were compared between patients with IPF and patients with non-IPF ILD according to corticosteroid dose. Categorical variables were compared using Pearson’s chi-square test and are reported as numbers and percentages. Continuous variables were compared using logistic regression models. Continuous variables with normal distributions are reported as mean and standard deviation, while variables with non-normal distributions are reported as median and interquartile range (IQR, 25th to 75th percentiles). We further investigated the relationship between clinical parameters and mortality using Cox proportional hazard models with stepwise selection of variables found to be significant in the univariate regression analysis.

Cumulative time-to-event distributions (survival) were estimated using the Kaplan–Meier method. Statistical significance was determined by the log-rank test. In all cases, *P* values < 0.05 were considered statistically significant. All statistical analyses were performed with IBM SPSS Statistics version 25.

## Supplementary Information

Supplementary Information.
