# Supplementary material for: Corticosteroid responsiveness in patients with acute exacerbation of interstitial lung disease admitted to the emergency department
Source: Sci Rep. 2021 Mar 11;11:5762. doi: 10.1038/s41598-021-85539-1 (PMC7952556; doi:10.1038/s41598-021-85539-1)
Supplement: Supplementary file 1 — Supplementary Information. [file 41598_2021_85539_MOESM1_ESM.docx]

**Corticosteroid responsiveness in patients with acute exacerbation of interstitial lung disease admitted to the emergency department**

Hye Jin Jang, MD; Seung Hyun Yong, MD; Ah Young Leem, MD, PhD; Su Hwan Lee, MD; Song Yee Kim, MD; Sang Hoon Lee, MD; Kyung Soo Chung, MD; Ji Ye Jung, MD, PhD; Young Ae Kang, MD, PhD; Young Sam Kim, MD, PhD; Joon Chang, MD, PhD; Moo Suk Park, MD, PhD*

**Supplemental material**

**e-Tables**

e-Table 1**.** Comparison of characteristics of survivors and non-survivors among IPF patients with acute exacerbation

| Variable | Total | Survivors | Non-survivors | *P*-value |
| --- | --- | --- | --- | --- |
| Total patients | 117 | 87 | 30 |  |
| Age, years | 69.4±9.9 | 70.3±9.2 | 66.8±11.5 | 0.10 |
| Sex, men | 94 (80.3) | 70 (80.5) | 24 (80.0) | 0.96 |
| Smoking exposure, No. (%) |  |  |  |  |
| Never | 48 (42.1) | 32 (38.1) | 16 (53.3) | 0.27 |
| Former | 64 (56.1) | 50 (59.5) | 14 (46.7) |  |
| Current | 2 (1.8) | 2 (2.4) | 0 (0.0) |  |
| Pack-years | 35.0 (20.0-50.0) | 37.5 (21.5-50.0) | 27.5 (18.0-38.0) | 0.34 |
| FVC % predicted | 57.0 (41.0-69.0) | 56.0 (42.0-69.0) | 58.5 (38.0-70.0) | 0.70 |
| FEV_1_ % predicted | 67.0 (50.5-80.0) | 67.0 (54.0-80.0) | 66.5 (46.0-80.0) | 0.55 |
| DL_CO_, % predicted | 44.0 (31.0-60.0) | 46.0 (36.0-63.0) | 35.5 (27.8-52.5) | 0.09 |
| RVSP (mm Hg) | 49.5 (37.0-61.5) | 48.0 (34.0-60.0) | 50.0 (37.0-70.0) | 0.27 |
| Initial P/F ratio | 220.0 (148.0-291.7) | 240.0 (158.0-308.3) | 203.8 (147.8-258.5) | 0.23 |
| Prednisolone (mg/kg) | 1.0±0.8 | 1.0±0.9 | 1.0±0.7 | 0.83 |
| Previous AE history | 43 (36.8) | 33 (37.9) | 10 (33.3) | 0.66 |
| Anti-fibrotics | 54 (46.2) | 44 (50.6) | 10 (33.3) | 0.95 |
| Supplemental O_2_ | 28 (23.9) | 20 (23.0) | 8 (26.7) | 0.14 |
| Prednisolone before AE | 49 (41.9) | 36 (41.5) | 13 (43.3) | 0.15 |
| Medical history |  |  |  |  |
| Hypertension | 25 (21.4) | 16 (18.4) | 9 (30.0) | 0.18 |
| Diabetes mellitus | 24 (20.5) | 20 (23.0) | 4 (13.3) | 0.26 |
| CAOD | 13 (11.1) | 11 (12.6) | 2 (6.7) | 0.27 |
| NTM | 4 (3.4) | 3 ( 3.4) | 1 (3.3) | 0.98 |
| Old TB | 8 (6.8) | 7 ( 8.0) | 1 (3.3) | 0.38 |
| COPD | 6 (5.1) | 5 ( 5.7) | 1 (3.3) | 0.61 |
| Malignancy | 21 (17.9) | 14 (16.1) | 7 (23.3) | 0.37 |
| CRP (mg/L) | 65.0 (21.0-136.0) | 60.0 (21.0-142.0) | 73.5 (21.0-136.0) | 0.46 |
| Need for mechanical ventilator | 24 (20.5) | 11 (12.6) | 13 (43.3) | < 0.001 |
| Use of vasopressors within 3 days | 19 (16.2) | 12 (13.8) | 7 (23.3) | 0.22 |

Abbreviation: IPF=idiopathic pulmonary fibrosis; FVC=forced vital capacity; FEV_1_=forced expiratory volume in 1 second; DLco=diffusing capacity of carbon monoxide; P/F ratio= partial pressure of oxygen in arterial blood (PaO_2_)/fraction of inspired oxygen (FiO_2_) ratio; GAP score system=gender (G), age (A), physiology (P); AE, acute exacerbation; CAOD=coronary artery occlusive disease; NTM=Non tuberculous mycobacterium; COPD=Chronic obstructive lung disease; Old TB=previous tuberculosis; CRP=C-reactive protein; Data are presented as mean, standard deviation, median, interquartile range, or frequency (%)

e-Table 2. Comparison of characteristics of survivors and non-survivors among non-IPF ILD patients with acute exacerbation

| Variable | Total | Survivors | Non-survivors | *P*-value |
| --- | --- | --- | --- | --- |
| Total patients | 65 | 55 | 10 |  |
| Age, years | 67.4±14.1 | 66.9±14.6 | 70.1±11.8 | 0.51 |
| Sex, men | 28 (43) | 24 (44) | 4 (40) | 0.83 |
| Smoking exposure, No. (%) |  |  |  |  |
| Never | 47 (78) | 40 (78) | 7 (78) | 0.96 |
| Former | 13 (22) | 11 (22) | 2 (20) |  |
| Current | 0 | 0 | 0 |  |
| Pack-years | 25.0 (20.0-40.0) | 25.0 (20.0-38.0) | 40.0 (0.0-40.0) | 0.64 |
| FVC % predicted | 67.0 (55.0-80.0) | 67.0 (54.0-80.0) | 60.0 (56.0-82.0) | 0.80 |
| FEV_1_ % predicted | 75.5 (61.0-92.0) | 76.0 (58.0-93.0) | 68.0 (61.0-90.0) | 0.74 |
| DL_CO_, % predicted | 49.0 (37.3-58.0) | 49.0 (38.3-58.0) | 42.0 (34.3-73.5) | 0.93 |
| RVSP (mmHg) | 39.0 (30.0-52.0) | 35.0 (30.0-50.0) | 41.5 (31.0-65.0) | 0.59 |
| Initial P/F ratio | 233.9 (179.6-339.3) | 220.7 (175.0-365.0) | 264.0 (188.0-295.4) | 0.90 |
| Prednisolone (mg/kg) | 1.1±0.9 | 1.20±1.0 | 0.8±0.3 | 0.16 |
| Previous AE history | 17 (26.2) | 15 (27.3) | 2 (20.0) | 0.57 |
| Supplemental O_2_ | 9 (13.8) | 9 (16.4) | 0 (0.0) |  |
| Prednisolone before AE | 42 (64.6) | 37 (67.3) | 5 (50.0) | 0.62 |
| Medical history |  |  |  |  |
| Hypertension | 8 (12) | 7 (13) | 1 (10) | 0.81 |
| Diabetes mellitus | 8 (12) | 7 (13) | 1 (10) | 0.81 |
| CAOD | 6 (9) | 6 (11) | 0 (0) | 0.27 |
| NTM | 3 (5) | 3 (5) | 0 (0) | 0.45 |
| Old TB | 3 (5) | 3 (5) | 0 (0) | 0.45 |
| COPD | 6 (9) | 6 (11) | 0 (0) | 0.27 |
| Malignancy | 15 (23) | 13 (24) | 2 (20) | 0.80 |
| CRP (mg/L) | 78.0 (27.0-140.0) | 72.0 (21.0-139.0) | 109.5 (77.0-183.0) | 0.20 |
| Need for mechanical ventilator | 11 (17) | 7 (13) | 4 (40) | 0.03 |
| Use of vasopressors within 3 days | 7 (11) | 5 (9) | 2 (20) | 0.31 |

Abbreviation: IPF=idiopathic pulmonary fibrosis; FVC=forced vital capacity; FEV_1_=forced expiratory volume in 1 second; DLco=diffusing capacity of carbon monoxide; P/F ratio= partial pressure of oxygen in arterial blood (PaO_2_)/fraction of inspired oxygen (FiO_2_) ratio; GAP score system=gender (G), age (A), physiology (P); AE, acute exacerbation; CAOD=coronary artery occlusive disease; NTM=Non tuberculous mycobacterium; COPD=Chronic obstructive lung disease; Old TB=previous tuberculosis; CRP=C-reactive protein; Data are presented as mean, standard deviation, median, interquartile range, or frequency (%)

**e-Figure legends**

e-Figure 1. Kaplan-Meier survival curves for comparing triggered AE-ILD (A) and non-triggered AE-ILD groups (B).


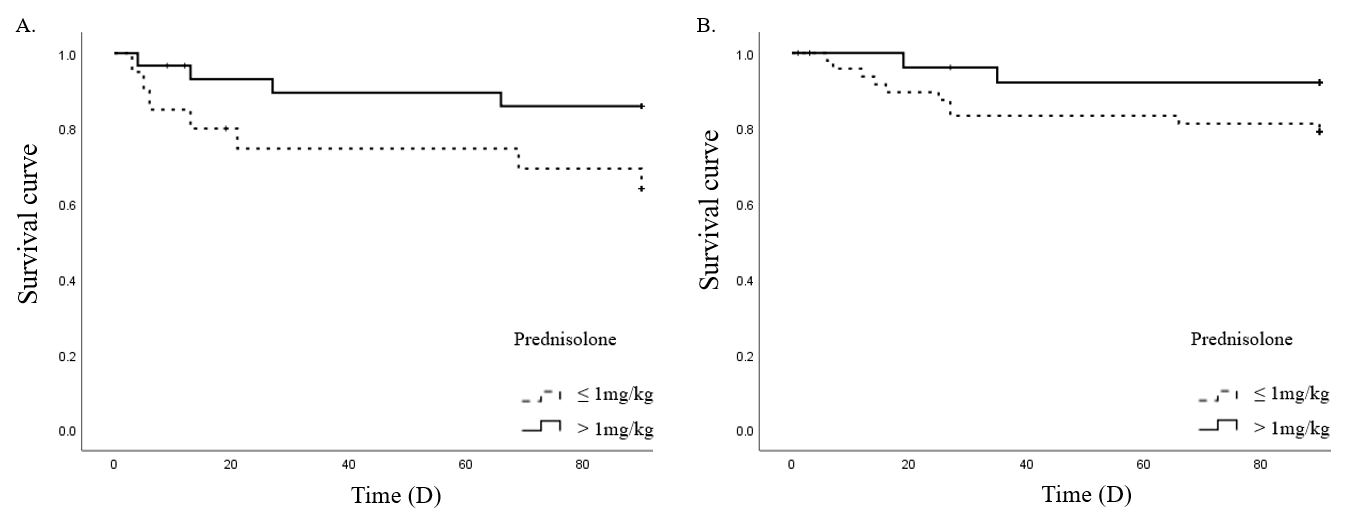


P=0.074


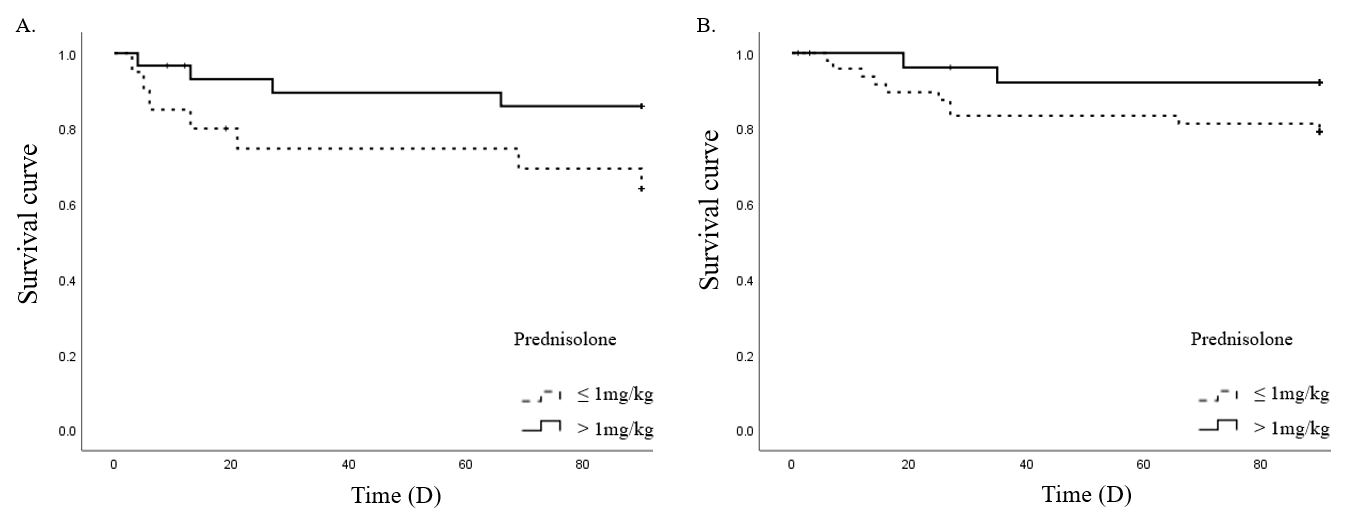


P=0.149
